# Developing a Novel Index for Individual-Level Social Determinants and Cardiovascular Diseases in the Coronary Artery Risk Development in Young Adults (CARDIA) Study

**DOI:** 10.3390/ijerph22030422

**Published:** 2025-03-13

**Authors:** Tao Gao, Yinan Zheng, Brian Joyce, Lei Liu, Lili Liu, Catarina Kiefe, Sarah Forrester, Bing Yu, Ankeet Bhatt, Penny Gordon-Larsen, Donald Lloyd-Jones, Kai Zhang, Lifang Hou

**Affiliations:** 1 Department of Preventive Medicine, Northwestern University Feinberg School of Medicine, Chicago, IL 60611, USA; 2 Center for Global Oncology, Institute for Global Health, Northwestern University Feinberg School of Medicine, Chicago, IL 60611, USA; 3 Division of Biostatistics, Washington University in St. Louis, St. Louis, MO 63110, USA; 4 Department of Population and Quantitative Health Sciences, University of Massachusetts Chan Medical School, Worcester, MA 01655, USA; 5 Department of Epidemiology and Human Genetics Center, School of Public Health, University of Texas Health Science Center at Houston, Houston, TX 77030, USA; 6 Division of Research, Kaiser Permanente San Francisco Medical Center, San Francisco, CA 94588, USA; 7 Department of Nutrition, Gillings School of Global Public Health, University of North Carolina at Chapel Hill, Chapel Hill, NC 27599, USA; 8 Preventive Medicine & Epidemiology Section, and Framingham Center for Population & Prevention Science, Boston University School of Medicine, Boston, MA 02118, USA; 9 Department of Population and Community Health, College of Public Health, The University of North Texas Health Science Center at Fort Worth, Fort Worth, TX 76107, USA

**Keywords:** social determinants of health, coronary artery calcification, left ventricular mass

## Abstract

Background: Social determinants of health (SDH) have been found to contribute to cardiovascular risk and the development of cardiovascular disease (CVD). However, few studies have examined early-life exposure to SDH and the combined effect of multiple SDH measures on CVD. There is an urgent need to develop an SDH index for use in practice and clinical settings. Methods: A total of 3189 participants from the CARDIA Study who had chest CT scans at the year-25 (Y25) visit were included in this study. Personal and parental SDH measures, including education, occupation, income, financial strain, and childhood family environment, were obtained through interviews. The participants’ coronary artery calcification (CAC) was measured using chest CT scans, and left-ventricular mass (LVM) was measured using M-mode echocardiography. The values of the individual social determinants of health (iSDH) index were determined based on individual-level measures and CAC using a supervised learning method—the Boosted Regression Tree (BRT) model. This index’s association with the LVM index (LVMI) was evaluated as an external validation using linear regression models adjusting for race, sex, BMI, smoking status, alcohol intake, and physical activity. Results: Each one-standard-deviation (SD) increase in the iSDH index was associated with an increase in LVMI ranging from 0.376 (95% CI −0.016, 0.767) at year 0 to 0.468 (95% CI 0.115, 0.821) at year 20. The association between the iSDH index and the LVMI was more pronounced as the participants aged. Also, the iSDH indices were more strongly associated with LVMI among Black participants (β = 0.969, 95% CI = 0.081, 1.858) than White participants (β = 0.202, 95% CI = −0.228, 0.633) at year 5. Conclusions: Higher iSDH indices in early adulthood were associated with increased LVMI values in midlife. The association between the iSDH index and CVD was stronger among Black adults than with White adults.

## 1. Introduction

Cardiovascular disease (CVD) is the leading cause of death in the US and worldwide, and it is a major cause of disability [1,2]. Traditional risk factors such as hypertension, dyslipidemia, diabetes, and tobacco use only explain a portion of the inter-individual differences in observed risk [3,4]. Social determinants of health (SDH) have long been known to be associated with CVD. Among the SDH factors, both individual (e.g., education, household income, and occupation) and parental factors (education and occupation) have been associated with CVD [5,6,7,8,9]. Studies have noted that SDH may play a significant role in racial health disparities in CVD [10,11,12].

Various methods have been proposed to integrate SDH and assess their relationship with adverse outcomes [13,14]. For example, the Social Vulnerability Index (SVI), created by the Centers for Disease Control and Prevention/Agency for Toxic Substances and Disease Registry (CDC/ATSDR) was derived from 15 census variables [13]. Also, the Area Deprivation Index (ADI) from the Neighborhood Atlas offers a measure of neighborhood disadvantage [14]. However, both indices are designed to assess risk at the community (rather the individual) level, and both have limited predictive ability for specific diseases when compared with total mortality [13,15]. Given the high burden of morbidity and mortality for cardiovascular diseases in both the US and worldwide, it would be very helpful to have a combined measurement of SDH for CVD prevention. By combining traditional regression models with machine-learning methods, a Boosted regression tree (BRT) model offers a better way to predict risk for diseases like CVD [16]. It accommodates complex linear and nonlinear responses to both community- and individual-level predictors while being relatively insensitive to collinearity problems [17].

With longitudinal measurements of social determinants of health during young adulthood and subclinical atherosclerosis measures in midlife, the Coronary Artery Risk Development in Young Adults (CARDIA) study offers a good opportunity to study the associations between individual social determinants of health (iSDH) and subclinical atherosclerosis as well as the racial differences between SDH and CVD [18]. Thus, we hypothesized that iSDH index values calculated from individual-level social determinants of health were positively associated with CVD. In this study, we aimed to quantify the association between the iSDH index and midlife subclinical cardiovascular disease using the left ventricular mass index (LVMI).

## 2. Methods

### 2.1. Study Population

CARDIA study included a longitudinal cohort of 5115 participants recruited from four urban areas in the US [18]. This study was designed to include participants balanced by sex, race, and education status within each center. After the first visit at year 0 (1985 to 1986), eight follow-up examinations were conducted through year 30 (2015 to 2016) [18,19]. In the current study, 3189 participants who attended the CARDIA study visit at year 25 (2010–2011) with chest CT scans were included, 2943 of whom were assessed in terms of LVMI. The institutional review boards at each participating institution approved the examinations, and written informed consent was given by all participants (ethical committee name: Northwestern University Institutional Review Board; approval code: STU00217160; approval date: 23 June 2022).

### 2.2. Individual Social Determinants of Health

Data on personal education, occupation, income, and financial strain were obtained through structured interview questionnaires at each visit. Parental education and occupation data were collected through an interview questionnaire at year 0. Factors related to adverse childhood family environments were ascertained through an interview questionnaire at year 15 [20]. Body weight was measured to the nearest 0.2 kg on a calibrated scale, whereas height was measured to the nearest 0.5 cm using a fixed vertical ruler. BMI was calculated as weight in kilograms divided by height in meters squared. Cigarette-smoking status was classified into three categories: never smoker, ever smoker, or current smoker. The CARDIA Physical Activity History questionnaire was used to estimate weekly physical activities over the past 12 months [21].

### 2.3. CT Measure of Coronary Artery Calcification

Multidetector computed tomography (CT) chest scans using a standardized protocol were performed for the participants in CARDIA to measure CAC at Y25 [22,23]. The CT images were measured by experienced image analysts at the central CT reading center located at Wake Forest University School of Medicine. The Agatston score was calculated using a dedicated computer workstation (TeraRecon (Tokyo, Japan)) corrected for slice thickness [24]. Information on the robustness of the CAC score has been published previously [25,26,27]. CAC presence was defined as CAC score > 0.

### 2.4. Left Ventricular Mass Index

Two-dimensionally guided M-mode echocardiography in a parasternal window was also performed on the CARDIA participants at the year-25 examination. All studies were recorded in digital format, and the recordings were later sent to the Johns Hopkins University Echocardiography Reading Center in Baltimore, Maryland [28,29,30]. Using standard offline image analysis software (version 3.2, Digisonics, Inc., Houston, TX, USA), experienced analysts made measurements based on the digitized images. The left ventricular mass index (LVMI) was acquired after dividing LVM by height raised to the power of 2.7 [31].

### 2.5. Statistical Analysis

Participants’ characteristics were summarized using means and SDs or counts and percentages for continuous and categorical variables, respectively. Boosted Regression Tree (BRT) models were used to regress CAC risk status (Yes/No) against individual social determinants of health to evaluate the contribution (weight and its direction) of each factor [16,17]. As a boosted version of the traditional regression tree model, the BRT model was constructed using the “gbm” function in the “dismo” package in “R (version 4.2.2)” software. In the BRT model, the learning rate and tree complex were set to 0.001 and 5, respectively, as in previous studies [16,17]. The contribution (relative influence) of each variable was scaled so that the sum added to 100 [16]. Then, each selected SDH, with its contribution (including the direction of the contribution) to CAC as obtained from the BRT models, was weighted and summed to develop the individual social determinants of health index [16] (Appendix A). We then standardized the index at each visit using the visit-specific standard deviation to make the effects more comparable among the study visits. We then evaluated the accuracy of the index with respect to CVD and assessed the association between the indices and year-25 LVMI values using multiple-linear-regression models. All models were adjusted for study center, age, sex, race, BMI, smoking status, alcohol consumption, and physical activity. All analyses (except for the BRT models, for which R version 4.2.2 was used) were performed using SAS (version 9.4, SAS Institute Cary, Cary, NC, USA). Two-sided tests were used throughout, and *p*-values less than 0.05 were considered statistically significant.

## 3. Results

Table 1 shows the results of descriptive analyses of the participants’ characteristics at the Y25 visit according to CAC status. Compared with the participants with CAC, the participants who were CAC-free at the Y25 visit tended to be younger (49.2 vs. 51.2 years old), female (65% vs. 35.5%), Black (49.7% vs. 42.2%), college-educated (78.2% vs. 72.9%), and have a household income of over USD 50K per year (65.8% vs. 60.6%).

Table 2 shows the contributions to (relative influence) and directions of individual-social-determinants-of-health factors at each visit with respect to the risk of CAC at year 25. Among these factors (for example, in year 15), personal income, an adverse childhood family environment, and personal education were the three that contributed most to the risk of CAC (15.75%, 14.22%, and 13.44% separately—when combined, they amount to about half of the total contribution), while in year 25, personal education instead of personal occupation contributed more to the risk of CAC (16.05% for participant education vs. 9.57% for participant occupation).

Table 3 shows the association between iSDH indices from year 0 to year 25 and LVMI at year 25. Each one-SD increase in the iSDH index is associated with an increased LVMI ranging between 0.376 (95% CI −0.016, 0.767) at year 0 and 0.468 (95% CI 0.115, 0.821) at year 20. As the subjects became older, the association between the iSDH index and left ventricular mass became stronger. Also, the iSDH index was more strongly associated with LVMI among the Black participants (β = 0.969, 95% CI = 0.081, 1.858), compared with the White participants (β = 0.202, 95% CI = −0.228, 0.633), at year 5 (Table 4).

## 4. Discussion

Our study is the first to integrate the various individual SDH, including personal, parental, and childhood environment, as a single SDH index and assess its association with midlife subclinical CVD (LVMI). In our study of healthy midlife adults, we found that adverse individual SDH factors were associated with an increased risk of CVD after adjusting for common lifestyle factors, including smoking, alcohol drinking, and physical activity. We found that the association between individual SDH index values and midlife CVD was stronger among Black adults compared with White adults, but we caution that this finding is not the same as the iSDH index being less important in the White population. The sample of Black people in CARDIA tended to have lower and less-variable SES in general, leading to a smaller variation in SDH factors. In this case, factors like genetics or lifestyle factors (instead of SDH) may play a greater role in the risk of CVD for White people than for Black people.

Studies have shown the effects of SES factors like education and income on CVD [32,33,34]. But since all of these factors interact with one another, it is difficult to assess the effect of just one factor while accounting for all of the others. On the other hand, BRT models can assess the contribution of each factor to CVD by including all the factors in the same model [17]. Also, the effect of SDH factors might change during the aging process and have a dynamic association with CVD. With the over 20 years of follow-up data in the CARDIA study, it is possible to assess this association from early adulthood to midlife. In our study, the association between iSDH indices and LVMI was stronger in midlife than in early adulthood. Also, during early adulthood, adverse childhood environments made the biggest contribution to CVD risk, while in midlife, personal education contributed more to the risk of CVD.

Black adults are at a significantly higher risk (1.6–2.4 times) of developing cardiovascular diseases than white adults [11]. Among the factors that affect CVD, individual-level SDH factors might play an important role. Our results showed that compared to the White population, the individual SDH index was associated with CVD risk to a greater degree in the Black population. Black individuals tend to have lower median incomes and are more likely to be unemployed and work low-paying jobs relative to their White counterparts [35]. Earlier studies showed that education and income gradients pertaining to health were weaker in the Black population than in the White population [36,37,38]. Studies have also found weaker associations with education and income among Black women, and education was a stronger predictor of CVD risk than income and occupation in the White population [38]. Also, structural and systemic factors, including residential segregation, may increase exposure to health-harming conditions (for example, air pollution, toxic waste, or other environmental hazards) to a greater degree for Black populations compared with White populations [39]. All of these factors might explain the racial differences in the association between the iSDH index and CVD.

The strengths of this study include the high-quality longitudinal data on SDH factors from CARDIA obtained from individuals from young adulthood through middle age. Furthermore, the balanced sampling of Black and White participants in CARDIA allows for race-specific analyses. However, our study has limitations as well. First, given the design of CARDIA, we were unable to study racial/ethnic minority groups outside of White and Black populations; thus, our results may not be generalizable to other populations. For example, studies have shown less consistent SES patterning in Hispanic populations compared with White populations [40,41]. Second, although CAC has been widely used as a diagnostic marker of atherosclerosis, studies have shown that about one-fourth to one-third of incident CVD events occur in those with a CAC degree of zero [42]. However, the association between the iSDH index and the LVMI (also a strong predictor of CVD events) in our study showed the validity of the iSDH index for the prediction of CVD. Third, there are other unmeasured confounding factors, such as diet and healthcare access, which may affect the associations between individual SDH factors and CVD. These factors might mediate the effect of individual SDH factors on CVD [43,44]. Further studies are needed to elucidate the functions of these factors. Other factors like pandemic events or environmental hazards might also reshape the influence of SDH factors on chronic diseases, including CVD, and require additional study [45,46].

## 5. Conclusions

In this study, we established an iSDH index that included personal, parental, and childhood-environment factors. Higher iSDH indices in early adulthood were associated with an increased LVMI in midlife. The association between the iSDH index and the LVMI was stronger in the Black population compared with the White population, which may reflect the fact that the iSDH index explained more of the variance in the Black population compared with the White population. Further studies should be conducted to explore the association with other chronic diseases and ethnicities other than the Black and White populations.

## Figures and Tables

**Table 1:** Characteristics of participants in CARDIA year-25 visit according to CAC status.

	Non CAC	CAC	*p*
	n = 2282	n = 907	
Age, mean (SD), y	49.7 (3.7)	51.2 (3.2)	<0.0001
Center, n (%)			
BHAM	532 (23.3)	203 (22.4)	
CHIC	504 (22.1)	213 (23.5)	
MINN	596 (26.1)	261 (28.8)	
OAKL	650 (28.5)	230 (25.3)	0.19
Race			
Black	1134 (49.7)	383 (42.2)	
White	1148 (50.3)	524 (57.8)	0.0001
Sex, n (%)			
Male	798 (35.0)	585 (64.5)	
Female	1483 (65.0)	322 (35.5)	<0.0001
Education			
High school or less	497 (21.8)	244 (27.1)	
Some college or more	1779 (78.2)	658 (72.9)	0.002
Personal income, n (%)			
≤USD 50 k	769 (34.2)	351 (39.4)	
>USD 50 k	1482 (65.8)	539 (60.6)	0.005
Financial strain, n (%)			
Not hard to pay for basics	1625 (71.5)	613 (68.1)	
Hard to pay for basics	648 (28.5)	287 (31.9)	0.06
BMI, mean (SD)	30.1 (7.1)	30.9 (7.2)	0.004
Alcohol, mean (SD), mL/day	10.6 (21.5)	14.9 (28.3)	<0.0001
Physical activity, mean (SD), total intensity score	326.2 (272.8)	357.4 (280.2)	0.004
Smoking status, n (%)			
Never	1448 (64.3)	460 (51.9)	
Former	483 (21.4)	208 (23.4)	
Current	322 (14.3)	219 (24.7)	<0.0001
Personal occupation, n (%)			
Farmers/Laborers	129 (6.4)	86 (10.7)	
Clerical/sales/housewives	710 (35.4)	265 (33.0)	
Executives/professionals	1167 (58.2)	452 (56.3)	0.0005
Adverse childhood family environment, n (%)			
No	974 (48.2)	399 (50.0)	
Yes	1045 (51.8)	401 (50.0)	0.43
Father’s education, mean (SD), y			
High school or less	1033 (54.2)	412 (55.3)	
Some college	870 (45.7)	333 (44.7)	0.64
Mother’s education, mean (SD), y			
High school or less	1214 (56.8)	488 (59.5)	
Some college	925 (43.2)	332 (40.5)	0.17
Father’s occupation, n (%)			
Farmer/Laborer	485 (25.6)	181 (23.3)	
Clerical/sales	409 (21.6)	154 (19.8)	
Executive/professional	999 (52.8)	443 (56.9)	0.15
Mother’s occupation, n (%)			
Farmer/Laborer	154 (7.1)	46 (5.4)	
Clerical/sales/housewife	1425 (65.6)	589 (68.6)	
Executive/professional	594 (27.3)	224 (26.1)	0.14
iSDH Index, mean (SD)	−0.03 (0.99)	0.15 (1.04)	0.006

**Table 2:** Contribution of Y0 to Y25 individual SDH for Y25 CAC estimate *.

ISDH Factors	Contribution (%)					
	Y0	Y5	Y10	Y15	Y20	y25
Parental						
Education of father	−9.61	−8.06	−10.43	−8.73	−9.06	−8.15
Education of mother	−14.35	−11.17	−13.68	−13.33	−8.02	−11.86
Occupation of father	−12.56	−12.78	−11.48	−11.75	−12.86	−10.19
Occupation of mother	−9.51	−7.84	−6.57	−9.23	−10.00	−8.58
Childhood						
Adverse family environment	17.62	17.43	16.27	14.22	8.72	14.43
Personal						
Personal education	−6.65	−9.01	−9.87	−5.96	−21.41	−16.05
Personal occupation	−7.81	−8.15	−11.69	−13.44	−8.10	−9.57
Personal income	−16.47	−13.97	−15.88	−15.75	−15.46	−14.41
Financial strain	−5.41	−11.58	−4.13	−7.60	−6.36	−6.77

* Contribution means the relative importance (%) of each predictor. The total contribution of all predictors is 100%.

**Table 3:** Linear-regression-based association between Y0-to-Y25 ISDH indices and Y25 left ventricular mass index (LVMI) *.

	Estimate	95% CI		*p*
ISDH_index_y0	0.376	−0.016	0.767	0.061
ISDH_index_y5	0.418	0.008	0.828	0.046
ISDH_index_y10	0.350	0.001	0.699	0.049
ISDH_index_y15	0.338	−0.003	0.679	0.052
ISDH_index_y20	0.468	0.115	0.821	0.009
ISDH_index_y25	0.378	0.038	0.717	0.030

* Adjusted for age, sex, race, center, BMI, alcohol drinking, smoking status, and physical activity.

**Table 4:** Linear regression for race-stratified associations for Y0-to-Y25 ISDH indices and Y25 left ventricular mass index (LVMI) *.

	Black	95% CI		*p*	White	95% CI		*p*
	Estimate				Estimate			
ISDH_index_Y0	0.817	−0.029	1.663	0.059	0.216	−0.198	0.630	0.307
ISDH_index_Y5	0.969	0.081	1.858	0.033	0.202	−0.228	0.633	0.357
ISDH_index_Y10	0.157	−0.519	0.832	0.650	0.423	0.018	0.828	0.041
ISDH_index_Y15	0.399	−0.235	1.033	0.218	0.285	−0.119	0.689	0.167
ISDH_index_Y20	0.586	−0.051	1.223	0.072	0.373	−0.052	0.798	0.086
ISDH_index_Y25	0.396	−0.214	1.006	0.204	0.320	−0.093	0.732	0.129

* Adjusted for age, sex, center, BMI, alcohol drinking, smoking status, and physical activity.

## Data Availability

A data-sharing agreement is required for use of all data, analytic code, and other materials used in this study. Approved investigators may access datasets, analytic code, and other materials via an analytic portal owned and administered by CARDIA Study.

## Appendix A. Method for the Calculation of the iSDH Index at CARDIA Visits

The BRT model was constructed using the “gbm” function in the “dismo” package in “R (version 4.2.2)” software. In the BRT model, the learning rate and tree complex were set to 0.001 and 5, respectively. Then, each selected SDH, along with its contribution (including the direction of the contribution) to CAC as obtained from the BRT models, was weighted and summed to develop the individual social determinants of health index. For the iSDH factors, father’s education, with ≤12 years of education coded as 0 and >12 coded as 1; mother’s education, with ≤12 years of education coded as 0 and >12 coded as 1; father’s occupation, either executive/professional or not (1/0); mother’s occupation, either executive/professional or not (1/0); adverse family environment, denoted as either an adverse family environment or a lack thereof (1/0); personal education, with years of personal education ≤12 coded as 0 and >12 coded as 1; personal occupation, either executive/professional or not (1/0); personal income, with a personal household income ≤50 k coded as 0 and >50 k as 1; and financial strain, denoted as whether it is difficult or not difficult to pay for basics (0/1). The calculated index was then standardized at each visit using the visit-specific standard deviation to make the effects more comparable among the study visits.
